# Unravelling the acute respiratory infection landscape: virus type, viral load, health status and coinfection do matter

**DOI:** 10.3389/fcimb.2024.1380855

**Published:** 2024-05-13

**Authors:** Hortense Petat, Sandrine Corbet, Bryce Leterrier, Astrid Vabret, Meriadeg Ar Gouilh

**Affiliations:** ^1^ University of Rouen Normandy, Dynamicure INSERM UMR 1311, CHU Rouen, Department of Pediatrics and Adolescent Medicine, Rouen, France; ^2^ University of Caen Normandy, Dynamicure INSERM UMR 1311, Centre hospitalo-universitaire (CHU) Caen, Department of Virology, Caen, France

**Keywords:** respiratory viral load, viral respiratory infections, respiratory syncytial virus, rhinovirus, respiratory viral co-infections

## Abstract

**Introduction:**

Acute respiratory infections (ARI) are the most common infections in the general population and are mainly caused by respiratory viruses. Detecting several viruses in a respiratory sample is common. To better understand these viral codetections and potential interferences, we tested for the presence of viruses and developed quantitative PCR (Polymerase Chain Reaction) for the viruses most prevalent in coinfections: human rhinovirus (HRV) and respiratory syncytial virus (RSV), and quantified their viral loads according to coinfections and health status, age, cellular abundance and other variables.

**Materials and methods:**

Samples from two different cohorts were analyzed: one included hospitalized infants under 12 months of age with acute bronchiolitis (n=719) and the other primary care patients of all ages with symptoms of ARI (n=685). We performed Multiplex PCR on nasopharyngeal swabs, and quantitative PCR on samples positive for HRV or/and RSV to determine viral loads (VL). Cellular abundance (CA) was also estimated by qPCR targeting the GAPDH gene. Genotyping was performed either directly from first-line molecular panel or by PCR and sequencing for HRV.

**Results:**

The risks of viral codetection were 4.1 (IC_95_[1.8; 10.0]) and 93.9 1 (IC_95_[48.7; 190.7]) higher in infants hospitalized for bronchiolitis than in infants in primary care for RSV and HRV respectively (p<0.001). CA was higher in samples positive for multiple viruses than in mono-infected or negative samples (p<0.001), and higher in samples positive for RSV (p<0.001) and HRV (p<0.001) than in negative samples. We found a positive correlation between CA and VL for both RSV and HRV. HRV VL was higher in children than in the elderly (p<0.05), but not RSV VL. HRV VL was higher when detected alone than in samples coinfected with RSV-A and with RSV-B. There was a significant increase of RSV-A VL when codetecting with HRV (p=0.001) and when co-detecting with RSV-B+HRV versus RSV-A+ RSV-B (p=0.02).

**Conclusions:**

Many parameters influence the natural history of respiratory viral infections, and quantifying respiratory viral loads can help disentangle their contributions to viral outcome.

## 1 Introduction

Multiple respiratory viruses can infect either concurrently or sequentially respiratory tracts, responsible for Acute Respiratory Infections (ARI) in the general population (Monto et al., 2014). Their impacts differ according to the populations’ profiles (age, geographic areas, industrialization, comorbidity, etc) (O’Grady et al., 2016). Viral codetections are defined as the detection of multiple viruses in the same patient sample at the same time. When several pathogens co-circulate in a patient, a cooperation or a competition between these pathogens may occur (Nickbakhsh et al., 2019). The frequency of viral codetections in patients with ARIs’ symptoms ranges from 25 to 70% in literature and may depend on numerous factors (Aberle et al., 2005; Martin et al., 2012; Kouni et al., 2013; Lim et al., 2017; Liu et al., 2021; Petat et al., 2021). According to a review of articles published in 2014 including studies about viral coinfections (Goka et al., 2014), 74% of hospitalized patients included in these studies were under the age of 6 years old. Studies including older patients or performed in primary care are very rare.

In hospital care, the two main viruses found in viral codetections are the Respiratory Syncytial Virus (RSV) and the Human Rhinovirus (HRV) (Petat et al., 2021): HRV/RSV codetections account for 11% of infants less than 2 years old hospitalized for fever or respiratory symptoms (Talj et al., 2022). RSV detection seems to be associated with a decreased probability for secondary HRV coinfection, suggesting a negative virus-virus interference (Greer et al., 2009). During an RSV infection, host could be refractory to other respiratory viruses. In a prospective cohort published in 2016, Karpinen et al. showed a negative association between HRV and RSV: HRV infection rates were lower in children who presented a recent RSV infection compared to children who did not (8% versus 14%, p<0,05) (Karppinen et al., 2016). Also, HRV is known to infect adults about 2 to 3 times every epidemic season (Sansone et al., 2013), and to cause exacerbations of chronic respiratory diseases and increase mortality in the elderly (Gerna et al., 2008; Jartti et al., 2015) (Longtin et al., 2010). RSV is known to generate ARIs in infants, being the main cause of acute bronchiolitis. Even if RSV is not responsible for a high mortality rate in developed countries, it still represents an important burden on health systems, during winter epidemics (Moore et al., 2020; Demont et al., 2021; Young and Smitherman, 2021). Viral loads of these viruses are not quantified in routine practice yet, and no technique is protocolized. Studies explored associations between severity of symptoms presented by the patients and the level of the respiratory viral loads (Jansen et al., 2011; Hartmann et al., 2022). Results are inconsistent between the studies, because of the heterogeneity of the patients (age, symptoms), the difference between primary or hospital care populations, the particularity of each respiratory virus, and the quantitation techniques.

While other respiratory viruses, such as influenza virus and RSV, cause destruction of airway epithelial cells, HRV is rarely associated with upper airway cytopathology. Using scanning and light electron microscopy of nasal biopsy samples from subjects with natural colds, Winther et al. found that the epithelial cells were detached; however, the epithelial cell lining and edges remained structurally intact (Winther et al., 1986). Similar preservation of cell morphology and composition has been observed for the nasal epithelium in studies of experimental HRV infection, where the extent of viral shedding did not correlate with the number of cells in the epithelium (Winther et al., 1986). Upper airway epithelium cytopathology is viral infection dependent and for some viruses, cellular abundance is not correlated with viral load, making CA normalization inappropriate. Precise assessment of respiratory damage would require characterization of cell types by immunological methods in respiratory samples.

In this context, we analyzed the respiratory samples of patients suffering from acute respiratory infections enrolled in two French cohorts (Guérande and ECOVIR). We performed the virological characterization and developed quantitative RT-PCR of RSV and HRV to determine viral loads (VL) and to test correlations between VL and demographic data, coinfections, cellular abundance and viral genotypes of these respiratory viruses.

## 2 Materials and methods

**Figure 1:**
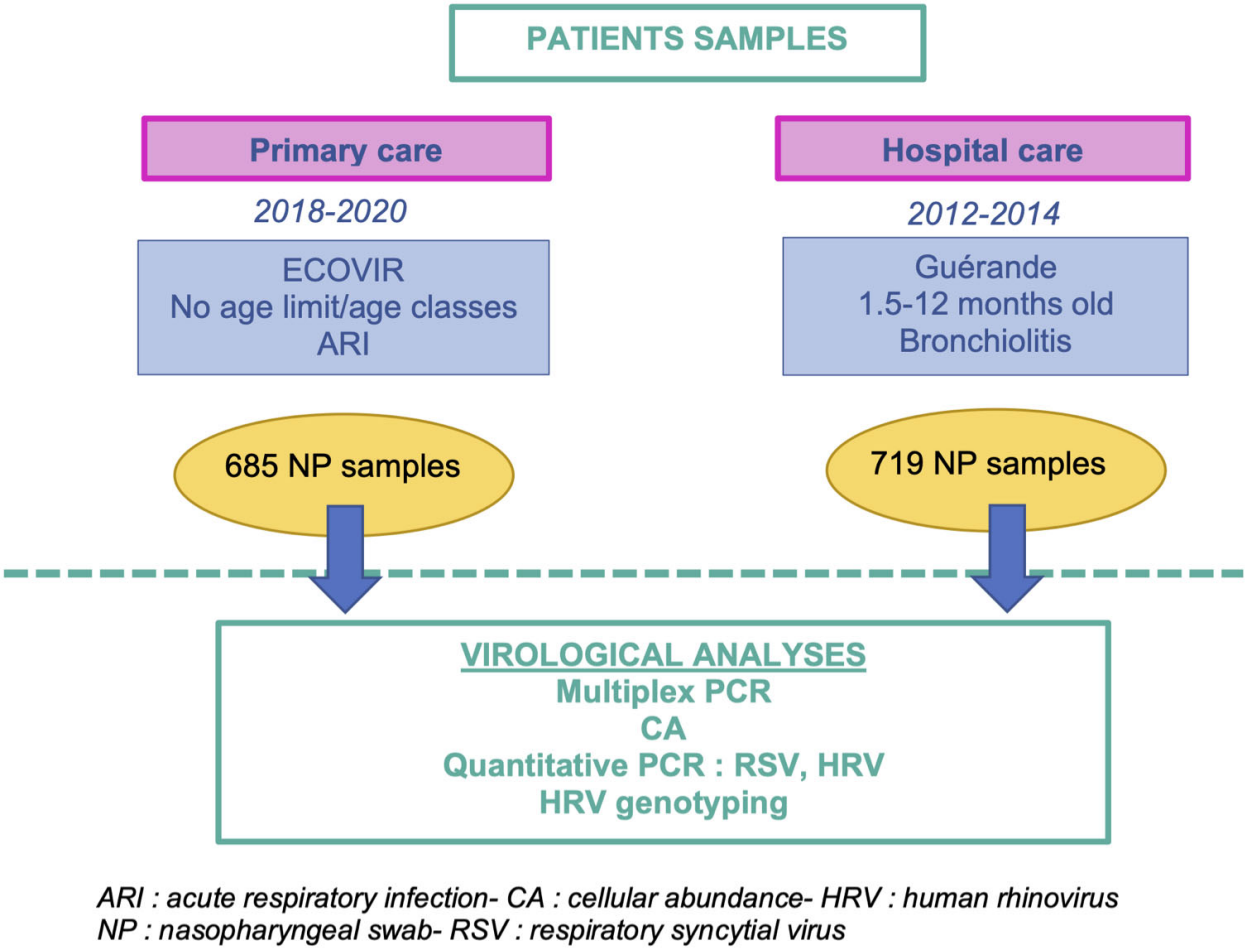
Workflow of the methodology of the study. This study is based on two cohorts of patients enrolled in hospital and primary care, Guérande and ECOVIR respectively.

Our analyses were based on two cohorts: Guérande and ECOVIR, in which nasopharyngeal swabs of patients suffering from acute respiratory infection were collected (**Figure 1**). In addition, results obtained from these cohorts were compared, whenever possible, to retrospective data obtained from the routine molecular diagnostic activity of the virology department of the hospital of Caen.

### 2.1 Cohort Guérande

Project Guérande was a prospective, double-blinded, multicentric study developed in France to test the efficacy of 3% hypertonic saline nebulization in acute viral bronchiolitis (Angoulvant et al., 2017; Petat et al., 2021). Briefly, 24 French hospital centers included patients between October 15, 2012 and April 15, 2014. Infants from 6 weeks to 12 months old who came to pediatric emergencies for a first episode of moderate to severe bronchiolitis were included and nasopharyngeal swabs were collected.

### 2.2 Cohort ECOVIR

Details on study design, laboratory methods, and bioinformatics were previously published (Petat et al., 2022). In short, project ECOVIR was a prospective, multicentric, non-interventional study developed in primary care in Normandy, France. ECOVIR was performed during two epidemic seasons (2018-2019, 12 weeks and 2019-2020, 21 weeks). For safety reasons and uncertainty regarding the early phase of COVID-19 emergence, the second season was abridged in March 2020 and it was not possible to relaunch it because of the lockdown of SARS-CoV2 pandemic. Patients were included by General Practitioner Investigators (GPI) during medical visits. Inclusion criteria were: French speaking people, whatever their age, consulting their general practitioner with symptoms of ARI. Each patient was informed, then examined, and a nasopharyngeal swab was performed. Data was collected by the GPI.

### 2.3 Biobank of hospitalized patients

During the 2016-2018 period, the virology department of the hospital center of Caen (CHU Caen) received 216 nasopharyngeal swabs from hospitalized patients. These samples were processed according to routine virological molecular analyses, and HRV genotyping was performed.

### 2.4 Ethics

For Guérande, written informed consent was obtained from the patients’ parents. The Saint-Germain-en-Laye Ethics Committee approved the study (reference 12020). For ECOVIR, we obtained the agreement of the “Est II” protection committee (study reference 18/10/10/63004) in January 2019. An information document was given to each patient included (non-opposition document). For retrospective data of the virology department of the hospital of Caen, sample processing was part of routine patient care and non-opposition consents for the result to be used for research, were obtained.

### 2.5 Molecular virologic techniques

#### 2.5.1 Extraction of nucleic acids

Every sample was achieved in Virology Laboratory of CHU Caen. Then nucleic acid (NA) were extracted with « QIAsymphony », (Qiagen, Hilden, Germany). To limit the number of thawing and therefore preserve the sample quality, we aliquoted NA extracts in 8μL microtubes.

#### 2.5.2 Viral detection by Nx-TAG RPP Luminex® and reverse transcription PCR for Rhinoviruses

NA were submitted to Nx-TAG RPP Luminex® kit for virologic identification and following manufacturer’s recommendations. Fifteen viruses (RSV-A, RSV-B, Rhinovirus/Enterovirus, Metapneumovirus, Adenovirus, CoV-HKU1, CoV-NL63, CoV-229E, CoV-OC43, Influenza, Para-Influenza 1, 2, 3, 4, Bocavirus) and 3 intra-cellular bacteria (*Chlamydophila pneumoniae, Legionella pneumophila, Mycoplasma pneumoniae*) were screened. Rhinoviruses were further genotyped by RT-PCR targeting the VP. The PCR mix was as follow: 10 µL of 2 X Reaction Mix (dNTPs + MgSO4), 0,8 µL of Superscript™ III RT/Platinum™ Taq Mix, 0,2 µL of RNAse inhibitor, 0,15 µL of each forward (5’ GGG ACC AAC TAC TTT GGG TGT CCG TGT 3’) and reverse (5’ GCA TCI GGY ARY TTC CAC CAC CAN CC 3’) primers. Eight μL of NA were then added and mixed thoroughly. Retro-transcription was performed at 50°C for 20 minutes, then activation of the polymerase enzyme was achieved by heating at 95° for 2 minutes. Then 35 cycles of PCR were composed of the three following steps: denaturation at 94°C for 15 seconds, hybridation at 63°C for 30 seconds, and polymerization at 68°C for 40 seconds.

#### 2.5.3 Development of real time RT-PCR

We created an aligned matrix comparing sequences of virus species (Rhinovirus A or B or C), forward and reverse primers, specific to each species, and “pan-rhino” primers matching all species. Primers were ordered dry and desalted at Sigmaaldrich company (https://www.sigmaaldrich.com/france.html).

**Figure 2:**
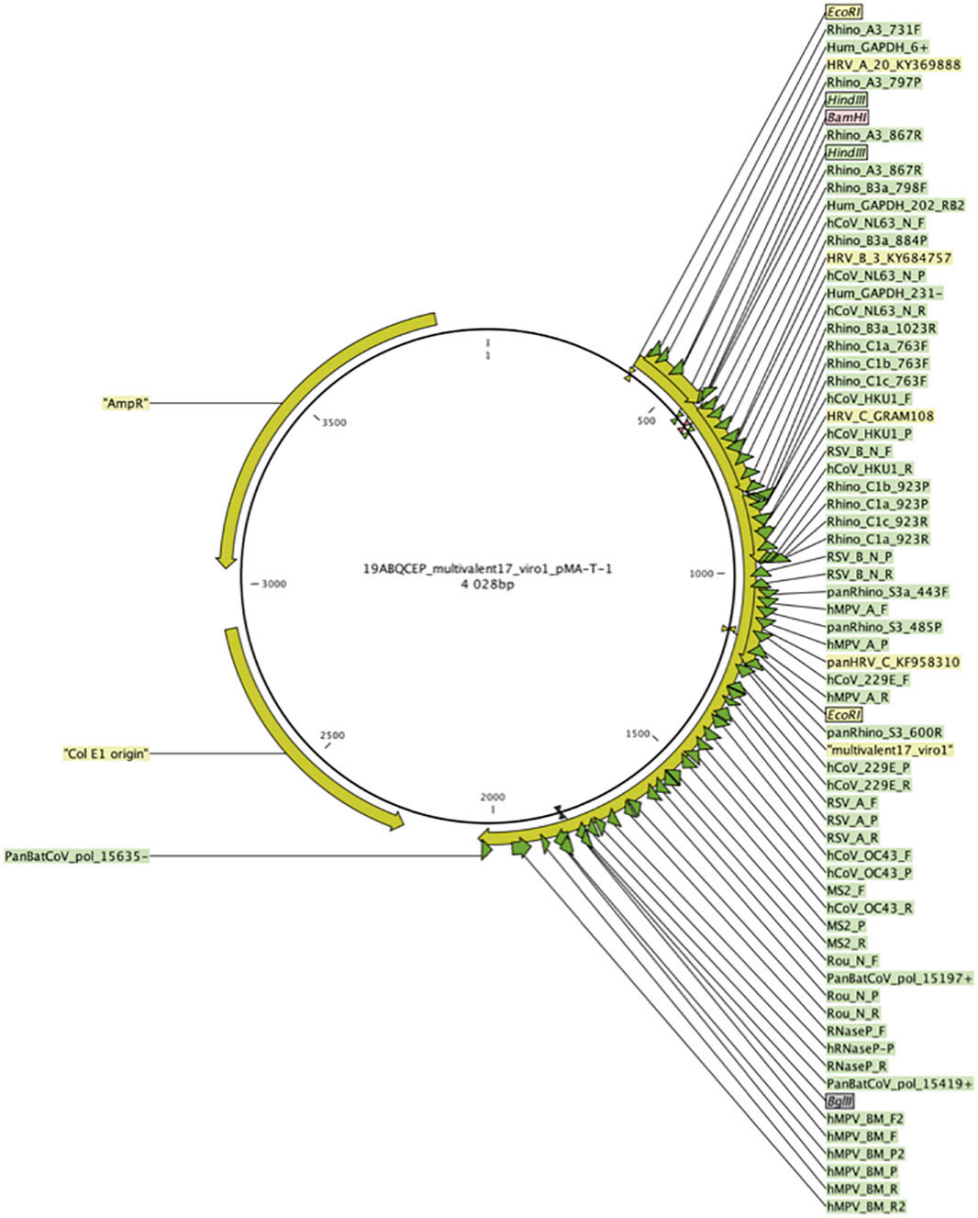
The plasmid "multivalent17_virol_pMA-T-1" used as a positive control and standard. Complementary regions to primers and probes used in detection systems designed in the study were combined in a pMA-T-1 plasmid backbone and designed to produce regions of comparable size of those targeted in the corresponding viral genome. Green arrows represent primer and probes complementary regions.

Universal positive control- In order to validate the PCR reaction, we designed a synthetic positive control multivalent plasmid “multivalent17_virol_pMA-T-1”. The HRV, VRS and GAPDH (Glyceraldehyde-3-phosphate dehydrogenase gene, used for human genome copy number estimation) primers, along with other primers used to detect respiratory viruses commonly used in our molecular diagnostic laboratory, were combined in a pMA-T-1 plasmid backbone to design the final universal positive control plasmid. Primers were placed at a distance producing an amplicon size comparable to the one of the PCR targets in the viral genome (**Figure 2**). The plasmid “multivalent17_virol_pMA-T-1” was ordered at Invitrogen Geneart (Thermo Fisher Scientific, France).

Amplification systems were validated on both the synthetic control plasmid and a reduced number of samples,. We used the same methodology to develop qRT-PCR of RSV-A and RSV-B. We performed qRT-PCR on « LightCycler 480 II » (Roche, Bâle, Switzerland) and used the « LightCycler^®^ 480 SV 1.5.1 » software. The PCR mix was as following: 10 µL of 2X Reaction mix, 0,4 µL of RNAse out, 0,4 µL of SuperScript III Platinum One-Step Quantitative RT-PCR System, 0,4 µL of each forward and reverse primers 50µM, 1 µL of probe 50µM. We used 16 µL of this mix with 4 µL of extract. The PCR program was as following: retrotranscription at 50°C for 15 minutes, enzyme activation at 95°C for 2 minutes and then 35 cycles of denaturation at 95°C for 10 seconds, hybridation at 50°C for 10 seconds, polymerization at 72°C for 20 seconds. Then we performed detection fluorescence.

Development of range of standards- Range of standards was obtained by performing dilution of quantified synthetic plasmid “multivalent17_virol_pMA-T-1”. Plasmid DNA was quantified by the « Qubit™ 4 Fluorometer » (Invitrogen, Carlsbad, California, USA), following manufacture’s recommendations and allowed us to calculate the number of copies.

Quantitation thresholds- We determined quantitation thresholds of the viruses by performing PCR in triplicates, on a range of dilution of the multivalent plasmid “multivalent17_virol_pMA-T-1” for HRV, RSV-A, RSV-B and GAPDH. Following thresholds were determined: 20 copies/μL for RSV-A, 4,5 copies/μL for RSV-B, 10 copies for HRV.

Viral and GAPDH quantitative tests on the complete collections- Once the systems had been validated, we performed qRT-PCR on every sample of the collection. Quantitation was computed with the software LightCycler^®^ 480 SV 1.5.1. Lineary regression was performed with the values of the range of standards and allowed us to calculate number of copies in samples. Analyses and graphical representation were performed with the R® software.

Cellular abundance- To measure cellular abundance, we quantified a cellular gene in every sample (GAPDH).

#### 2.5.4 Rhinovirus sequencing

RT-PCR products were loaded on agarose gel for electrophoresis, and samples positive to rhinovirus VP region were selected for sequencing. Agarose gel was prepared with 1.8g of agarose with 120mL of TBE (Tris Borate EDTA) 1X, and 10µL of GelRed™ Nucleic Acid Gel stain. Migration was performed for 30 minutes at 100V. RT-PCR products producing visible band on the gel were visualized with « Molecular Imager^®^ GelDoc™ XR+ Imaging System », (BioRad, Hercules, California, USA) and ImageLab® software. Amplicons (5μL) were digested (clean-up) with 2μL of ExoSAP-IP (ThermoFischer, Waltham, Massachusetts, USA), during 15 minutes at 37°C followed by inactivation of enzymes at 80°C for 15 minutes in a thermocycler. Sequencing reaction was performed with the kit « BigDye Terminator version 1.1 cycle sequencing kits » following manufacturer’s recommendations (Applied Biosystems, Foster City, California, USA). Then samples were purified by sephadex gel column filtration, « Séphadex ^®^ G50 », following manufacturer’s procedure (Sigma, Saint-Louis, Missouri, USA). Sequencing was then performed with Sanger’s method on « Applied 3500 Genetic Analyzer » (Applied Biosystems, Foster City, California, USA). A bioinformatic analysis was handled with the « CLC Main WorkBench 8 » software (CLC BioQiagen^®^, Hilden, Germany). Then the sequences were submitted to blast analysis (https://blast.ncbi.nlm.nih.gov/Blast.cgi) and phylogenetics reconstruction methods.

### 2.6 Statistical analysis

Statistical analyses were performed with standard methods using RStudio software (version 1.1.456). Univariate, descriptive statistics and graphs were prepared using Excel© (Microsoft) and RStudio© softwares. All significance tests were 2-tailed (0.05). For normally distributed data, Student’s *t* test was used. To compare means of different groups, Anova test was implemented with RStudio software. For non-normally distributed data, Kruskal-Wallis test was used to highlight a group effect on measured viral loads among infection groups (mono or coinfection). If a group effect was found, then Dunn’s post-tests were performed to identify which groups significantly distinguish from another. Due to multiple tests, p-value were adjusted using the Benjamini-Hochberg method.

### 2.7 Study limitations

Unfortunately, concerning ECOVIR, we had to stop inclusions of patients during the second season of ECOVIR, for safety reasons and uncertainty regarding the early phase of COVID-19 emergence. It was not possible to relaunch it because of the lockdown of SARS-CoV2 pandemic. It should be noted that cellular abundance value may be affected by biases because of the technic of sampling (naso-pharyngeal swab), a manual gesture performed by numerous GPIs participating in the study. Sample delay, defined by the number of days between the first symptoms and the day of sample, can be biases because based on patient declarations.

## 3 Results

Seven hundred and nineteen samples came from project Guérande, 685 patients were included in project ECOVIR (191 during the first season, 494 during the second season). Moreover, this “real life” study is inherently multifactorial (patients suffering from respiratory infections with numerous types of viruses, complex viral/viral and viral/host interactions, inter-individual variations). Consequently, it was sometimes difficult to overcome sample size effect (most co-infection types do have a too small sample size to be statistically evaluated) and to evaluate precisely potential confounding effects.

### 3.1 Viral prevalence, codetections and cellular abundance

Qualitative results of Multiplex PCR were very different for both cohorts (Guérande, ECOVIR, **Table 1A**). In ECOVIR, negative samples were less frequent in younger ages, and represent almost 50% of samples in the elderly (>65 years old). Thirteen percent of samples were positive to RSV in infants and in the elderly in primary care, versus 90% in infants in hospital. RSV was co-detected in 56% of cases in infants in hospital, but only in 24% of cases in primary care. HRV was co-detected in about 30% of cases in both cohorts (Petat et al., 2023).

Table 1Virological results.

**A.** Multiplex PCR using NxTAG Luminex Panel.

Viruses	Guérande- n=719 n (%)	ECOVIR- n=685 n (%)
RSV-A	441 (61)	27 (4)
RSV-B	297 (41)	11 (1.6)
HRV	246 (34)	193 (29)
CoV-229E	5 (0.6)	12 (1.8)
CoV-OC43	19 (3)	15 (2.2)
CoV-HKU1	4 (0.5)	17 (2.5)
CoV-NL63	10 (1)	17 (2.5)
Flu	4 (0.5)	98 (14.5)
PIV	59 (8)	34 (5)
BoV	49 (7)	7 (1)
hMPV	41 (6)	32 (4.7)
ADV	55 (8)	10 (1.5)
Negative	12 (2)	221 (32.7)

**B.** Viral codetections.

Detected virus	Guérande (n=719) n (%)	ECOVIR (n=685) n (%)	p	OR CI_95%_
RSV	263 (56)	9 (24)	<0.001	4.1[1.8 ; 10.0]
HRV	229 (93)	27 (14)	<0.001	93.9 [48.7 ; 190.7]
CoV	34 (92)	7 (12)	<0.001	75.6 [17.7 ; 482.5]
Flu	4 (100)	5 (6)	<0.001	NA

RSV, respiratory syncytial virus; HRV, human rhinovirus; CoV, coronavirus; Flu, influenza; PIV, parainflmuenza; BoV, bocavirus. The OR values are calculated referred to the Guérande’s group for each virus.

**Figure 3:**
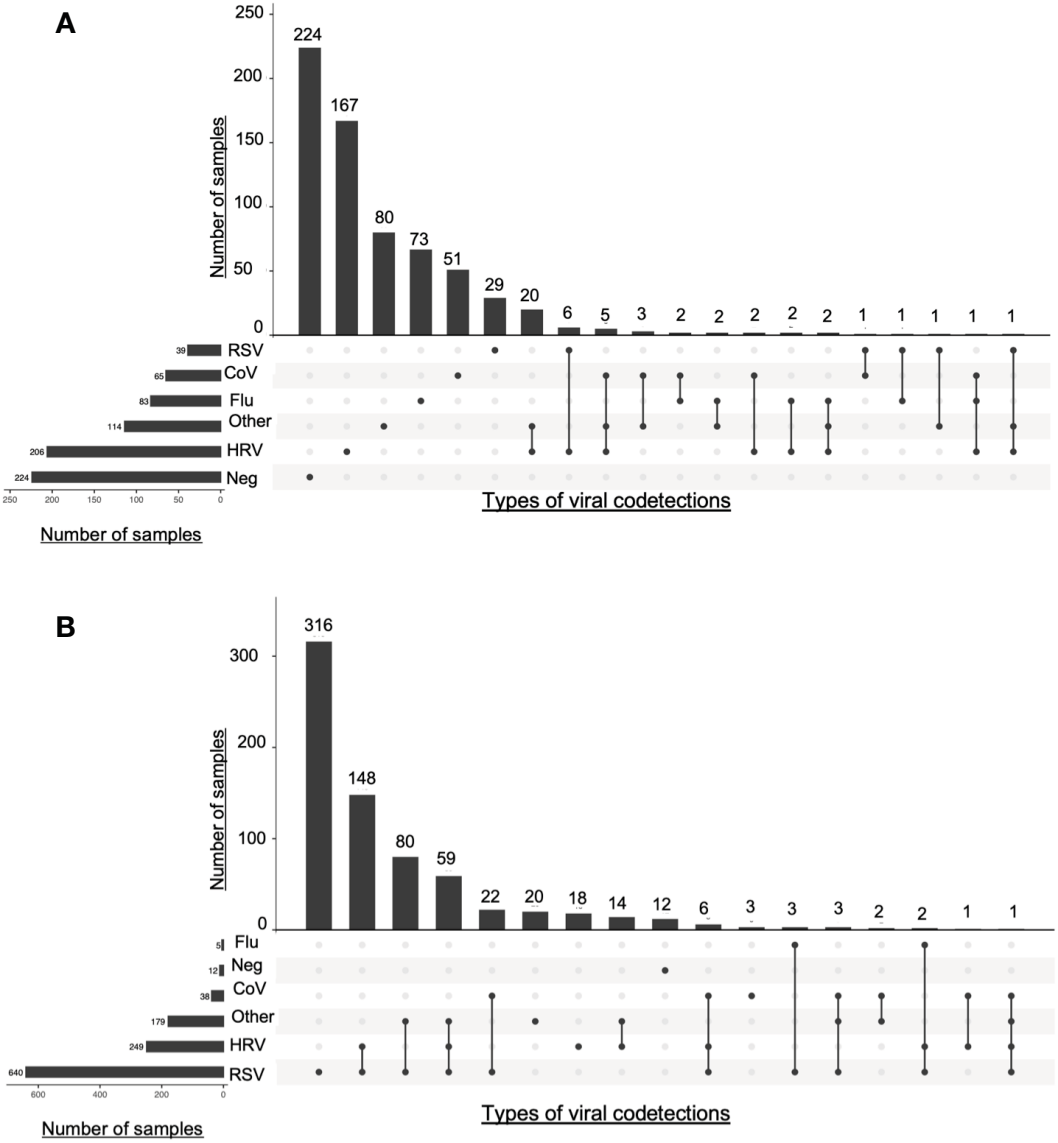
Intersections of viral detections in Guerande and ECOVIR cohorts. The number samples of each viruses is represented on the left side. At the top of the figure are represented the numbers of codetections described under. Lines linking dots represent intersection of viral detections or codetections**(A)** ECOVIR; **(B)** Guérande.

Viral codetections rates were 55% and 7.6% in Guérande and ECOVIR respectively (**Table 1B**). The risks of codetection were 4.1 and 93.9 higher in hospitalized infants than in primary care for RSV and HRV respectively (p<0.001). Repartition of viral codetections in primary care was highly variable in the two cohorts (**Figure 3**). The most abundant codetection was HRV/RSV-A. No codetection was observed in samples positive to RSV-B in primary care. Influenza virus was detected with another virus in only 6% of total influenza virus positive samples.

**Figure 4:**
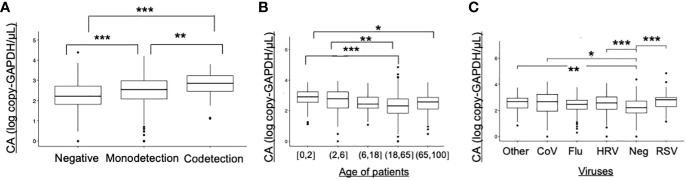
Cellular abundance in ECOVIR (primary care) samples. **(A)** Cellular abundance and viral codetections; **(B)** Cellular abundance according to the age of patients; **(C)** Cellular abundance according to the viruses found in samples Statistical test: Anova test *p<0.05, **p<0.01; ***p<0.001.

Cellular abundance (CA) in samples with viral monodetection was significantly higher than in negative samples (p<0.001), and significantly lower than samples with viral codetections (p<0.001) (**Figure 4A**). CA were significantly higher in younger patients under 6 years old compared to the adults aged between 18 and 65 years (p<0.01, **Figure 4B**). Compared to the negative samples, CA were significantly higher in samples positive to RSV (p<0.001), HRV (p<0.001), or CoV (p<0.05) (**Figure 4C**).

### 3.2 Respiratory viral loads

#### 3.2.1 Viral loads and age classes in ECOVIR (primary care)

**Figure 5:**
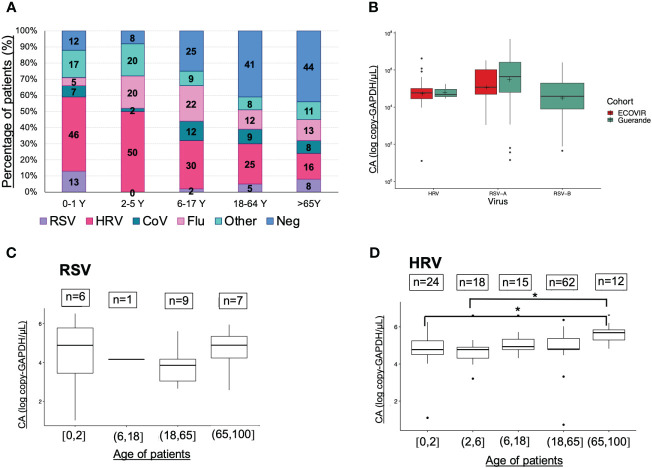
Viruses prevalences, viral loads (VL) and age classes. **(A)** Viruses and age classes (cohort ECOVIR); **(B)** RSV-A, RSV-B and HRV VL in both cohorts (Guérande and ECOVIR); **(C)** RSV VL and age of patients (cohort ECOVIR); **(D)** HRV VL and age of patients (cohort ECOVIR). *p<0.05 VL are expressed in number of copies per microliter of sample.

Results from ECOVIR were used to compare the viruses’ viral loads between all ages of population (**Figure 5A**). HRV represents almost a half of samples in children under 6 years old, and only 23% in adults (more than 18 years old). RSV was detected in 13% of infants (0-1 year old), very rare in children between 2 and 18 years old, and represents only 5% of samples in adults from 18 to 65 years old. In the elderly, the rate increased to 8%. RSV-A and RSV-B VL tend to be higher in hospitalized patients compared to patients in primary care, but not HRV VL (**Figure 5B**).

We compared respiratory VL in samples from ECOVIR cohort, because all ages were represented. The number of samples per age class was low and no significant difference was found between them concerning RSV VL (**Figure 5C**). Nevertheless, a tendency toward a higher RSV VL was observed in infants and the elderly compared to the adult population. Regarding HRV, VL in age classes 0-2 and 2-6 years were significantly lower compared with VL in age class >65 years old (p= 0.04 and 0.02 respectively) (**Figure 5D**). We compared VL in Guérande and in infants from ECOVIR (same age population): RSV-A VL was higher in hospitalized infants (p=0.05), HRV VL was higher in infants in primary care (p=0.005), and there was no difference concerning RSV-B VL.

#### 3.2.2 Viral loads and mono or co-detections in Guérande (Hospitalized infants)

**Figure 6:**
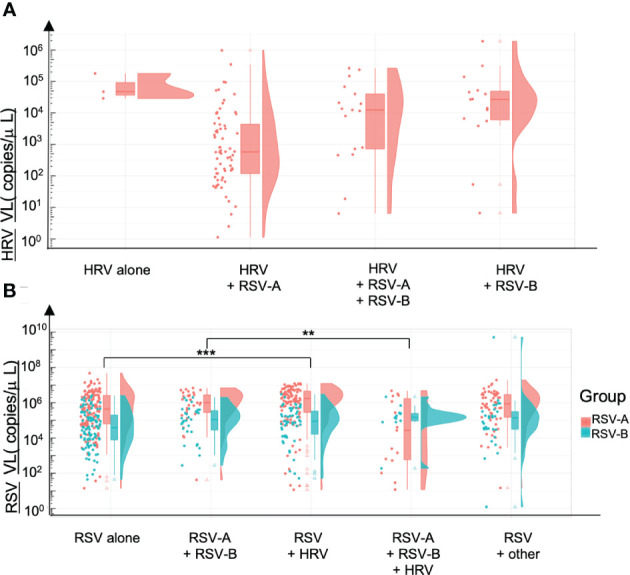
Viral loads and codetections of HRV and RSV. Viral loads are expressed in number of copies per microliter of sample. **(A)** HRV viral loads alone and in coinfection with either RSV-A, RSV-A & RSV-8 or RSV-B. **(B)** RSV-A and RSV-B viral loads alone or in coinfection with RSV (B or A) or HRV or other viruses. **p<0.01; ***p<0.001.

We compared HRV and RSV VL according to the codetections found, in the Guérande’s cohort, because of the high rate of viral codetections, and of the homogeneity of the cohort. HRV VL is higher when detected alone compared with samples co-detected with RSV-A, with RSV-B and with RSV-A and RSV-B, but not significantly (p=0.309) (**Figure 6A**). We can also note that RSV-A tends to replicate more than RSV-B. A significant increase of RSV-A VL was found when codetected with HRV only (p=0.001). At the opposite, a significant decrease of the RSV-A VL was observed when codetected with RSV-B + HRV, compared with RSV-B (p=0.02) (**Figure 6B**). No difference of VL was observed between RSV-B found alone and in codetections (p=0.90).

#### 3.2.3 C. Viral loads and sample delays

**Figure 7:**
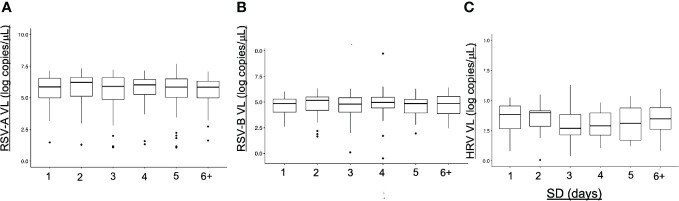
Viral loads (VL) and sample delays (SD). Viral loads are expressed in number of copies per microliter of sample. Sample delays are based on patient declarations and represent the number of days separating the onset of symptoms and the nasopharyngeal sampling. No difference between the SD was noted for each virus. **(A)** SD for RSV-A **(B)** SD for RSV-B **(C)** SD for HRV.

Sample delay was defined as the number of days between the onset of symptom and the nasopharyngeal swabbing. To propose a comparison with least biases possible, VL of samples from a unique cohort were considered (Guérande, accounting for most homogeneous samples with a better precision of sample delay estimation). There was no significant difference between VL according to the samples delays for HRV and RSV (**Figure 7**). For HRV, we can notice a trend toward a higher HRV VL during the first two days of symptoms.

#### 3.2.4 Viral loads and cellular abundance

**Figure 8:**
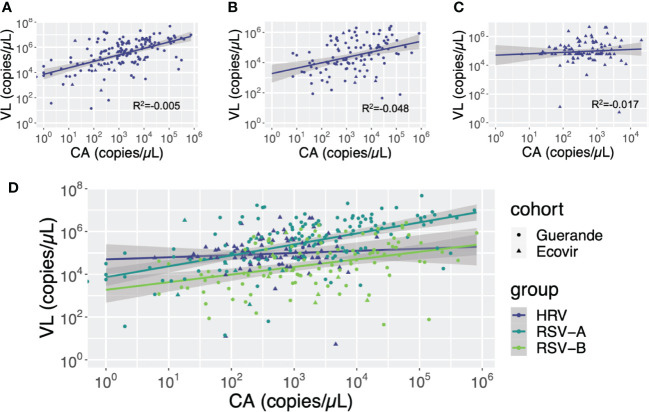
Correlation between cellular abundance (CA) and viral loads (VL) according to viruses. Cellular abundance and viral loads are expressed in copies of GAPDH gene per microliter and copies of the viral gene per microliter, respectively. **(A)** RSV-A: **(B)** RSV-B: **(C)** HRV: **(D)** HRV, RSV-A and RSV-B.

Cellular abundance was significantly lower in negative samples than in positive sample, regardless the virus (p<0.001). Despite VL and CA are not correlated, we see important variation in CA, with age or according to virus type (**Figure 8**). The strongest linear dependency is noted for HRV first and then RSV-A while RSV-B viral loads seem much less associated with cellular abundance, highlighting notable differences between viruses.

### 3.3 HRV genotyping

**Figure 9:**
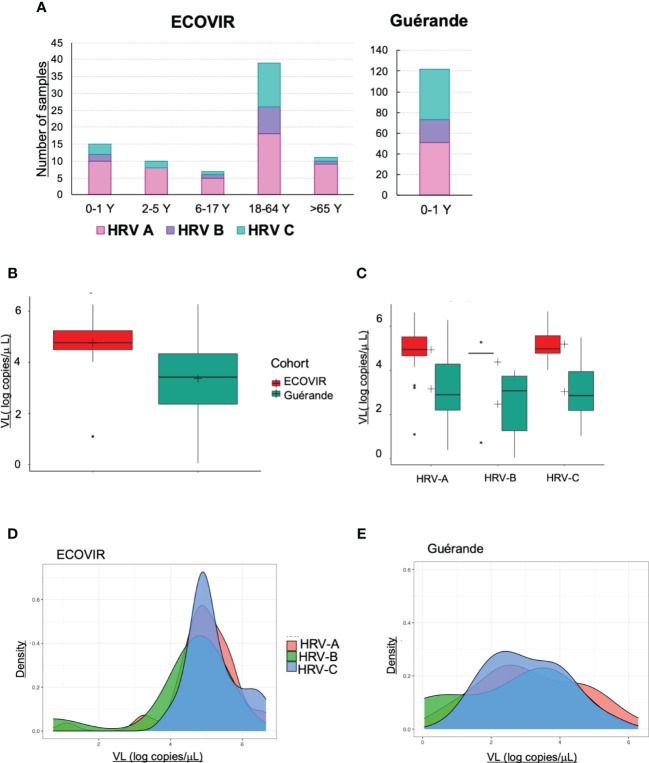
HRV. **(A)** Number of samples of HRV-A, HRV-B and HRV-C according to the age of the patients; **(B)** HRV VL in infants in ECOVIR and in Guérande; **(C)** HRV-A, B and C VL in ECOVIR and Guérande; **(D)** density plot of HRV-A, B and C VL in ECOVIR; **(E)** densityplot of HRV-A, B and C VL in Guérande.

PCR Multiplex tests found 246 (34%) and 193 (29%) samples positive to HRV/enterovirus respectively in Guérande and ECOVIR. HRV genotyping was performed on positive samples: we found respectively 61, 15 and 24% in ECOVIR (n=82) and 42, 18, and 40% in Guérande (n=122) of HRV-A, HRV-B and HRV-C (**Figure 9A**). Viral loads were determined on 220 and 131 samples for Guérande and ECOVIR respectively. HRV VL was significantly higher in infants from primary care compared to hospitalized infants (**Figure 9B**). All HRV VL were significantly higher in primary care compared to hospitalized patients (respectively p= 0.04, 0.008 and 0.03 for HRV-A, HRV-B and HRV-C) (**Figure 9C**). No significant difference was found between VL according to HRV serotypes in both cohorts (p=0.099). HRV VL were more variable in Guérande compared with ECOVIR cohort (**Figures 9D, E**).

**Figure 10:**
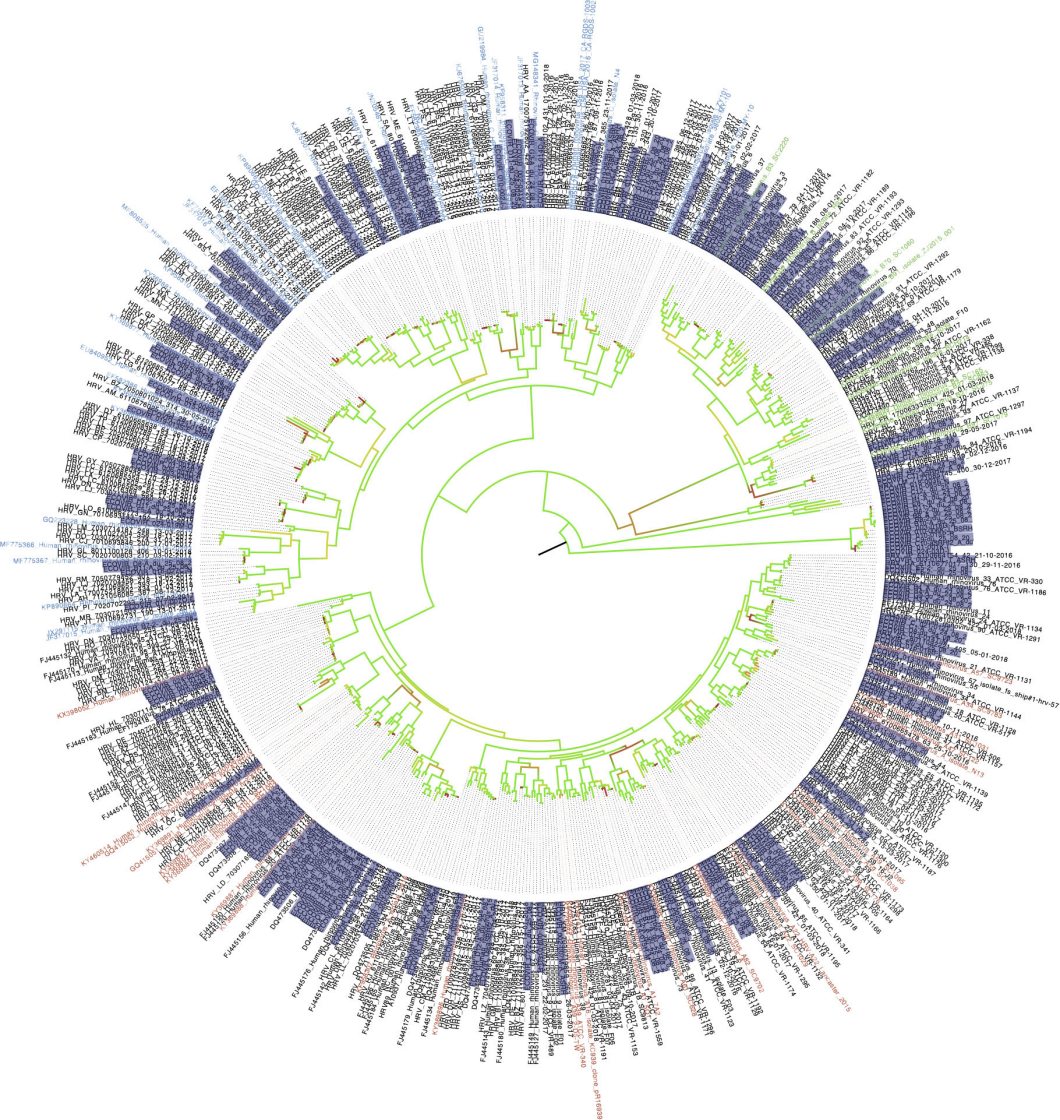
Phylogenetic tree of HRV in samples. ECOVIR and Guérande samples are highlighted in purple. Blue, green and red name indicate Rhinovirus A, B and C respectively (nbci data). Black names starting with a reference number represent data from ncbi database. Black names starting with "HRV" refer to data obtained from routine virology department of university hospital of Caen surveillance. In red come from ospital routine's patients; samples in black are the references Genbak.

We constructed the phylogenetic tree of HRV found in the samples from both cohorts (**Figure 10**). According to phylogenetic analysis, ECOVIR samples accounted for representative and balanced diversity of each HRV genotype.

## 4 Discussion

Within this work, following viral characterization, viral codetections in ARI were explored by quantifying respiratory VL of viruses mostly detected (RSV and HRV) in nasopharyngeal swabs. These samples came from two distinct and different cohorts: one performed in hospital in infants with bronchiolitis (Guérande), and the other one in primary care in patients of all ages mostly free of comorbidity (ECOVIR). The Guérande cohort confirm the association of bronchiolitis with RSV but this virus also circulates in the general population as shown by ECOVIR’s results. These cohorts also confirm the preferential circulation of respiratory viruses in childhood and the importance of RSV and HRV in respiratory health in childhood and elderly. These differences have previously been extensively discussed in Petat et al. in 2021. Similarly to previous studies, we found a higher prevalence of HRV-A, then HRV-C compared to HRV-B, highlighting differences in the epidemiology of the viruses.

First, in line with literature, we found that the prevalence of codetections is related to the age of patients with ARI. Young age is associated to higher codetection rates in the general population (Nickbakhsh et al., 2016; Heimdal et al., 2022). The high likelihood of coinfections in childhood may be attributable to the combination of the relatively synchronous seasonal epidemiology of most respiratory viruses and immature immunity of children, making them more susceptible to respiratory viruses during their first winter of life. Other studies including children under 5 years of age reported higher frequencies of viral codetections than those including adolescents and young adults (Nickbakhsh et al., 2019). A study published in 2016 analyzing respiratory specimens from 44230 respiratory infections between 2005 and 2013 showed that viral codetections were significantly more common in patients younger than one year (Nickbakhsh et al., 2016). Some viruses may be more represented than others in these codetections. In our study, RSV was co-detected in 56% of hospitalized infants presenting bronchiolitis, but only in 30% in the same group in primary care. That is what is described in literature: the rate of viral coinfections appears to be greater in inpatient versus outpatient cohorts (Aberle et al., 2005; Calvo et al., 2008; Libster et al., 2010). This suggests that viral codetections could also be associated with clinical severity of patients. Our team previously reported that there was a greater severity of respiratory infection in infants hospitalized for bronchiolitis infected with RSV alone compared to those infected with HRV alone (Marguet et al., 2009). HRV is the respiratory virus the most frequently found, in all ages, in all populations. We know thanks to studies performed in general population that adults are infected by HRV several times yearly (Monto et al., 2014). In a previous study, we found that, in infants, the risk of being infected by HRV is higher in the absence of RSV, suggesting interferences or exclusion mechanisms between these two viruses (Petat et al., 2021). We found the most frequent codetection in primary care was HRV/RSV-A, and that RSV-B was never co-detected, but the number of RSV-B positive samples was low (n=11) and the lower circulation of RSV-B may partially explain this result. This is different from data we found before in the Guérande’s cohort (hospitalized patients) (Petat et al., 2021), where RSV-B was co-detected in 63% of cases. HRV/RSV codetection is the most frequent described codetection in studies (Nickbakhsh et al., 2016; Nickbakhsh et al., 2019), even if influenza viruses, coronaviruses and parainfluenza viruses are implicated too. However, RSV detection is associated with a decreased probability of HRV coinfections, indicating a negative virus-virus interaction (Greer et al., 2009; Martin et al., 2013). Moreover, in a 2016 prospective cohort study, Karppinen et al. showed a negative association between RSV and HRV in infants younger than 24 months: the rate of HRV infections was lower in children who had a recent RSV infection compared to children who were not infected (8% versus 14%, p<0.05) (Karppinen et al., 2016). This is in line with our results and suggests that during or in the immediate aftermath of RSV infection, the host may be refractory to other viruses, maybe because of the local innate immunity activation and inflammation in response to infection. In a study published in 2020, based on *in vitro* co-infections, Essaidi et al. showed that flu and RSV could interfere with HRV replication, whereas HRV does not interfere with either of these viruses (Essaidi-Laziosi et al., 2020). This result is in line with our results regarding the apparent lower replication of HRV in coinfection with RSV. Essaidi et al. also suggested a key role of IFN production to explain these interferences. Other experiments are now awaited to confirm these hypotheses and detail the mechanisms of interferences.

We performed molecular estimation of cellular abundance (CA) in samples. A higher CA was found in samples with viral codetections compared to samples with monodetection. The lowest CA was found in negative samples. CA is probably multifactorial and may reflect a combination of desquaming cells, airway macrophages and other innate immunity recruited cells and lysed cells directly resulting from the replication of viral viruses in the respiratory tract (Barreto-Duran et al., 2022). We noted that CA was higher in children under the age of six. So, cell lysis and inflammatory response could be more intense in these infants. At the opposite, lower CA in the elderly may be explained by relative immune senescence and a weaker innate immune response at this age. Samples positive to RSV, HRV and CoV also presented a higher CA than negative samples, contrary to samples positive to influenza viruses. This may highlight significant differences between viruses in their ability to exploit cell machinery at their own advantage, to avoid or control host’s innate immune response. Nevertheless, while VL and CA are not correlated, viral-induced lysis or differential cell recruitment in response to inflammation in ARI both contribute to CA in the course of the infection. A most in-depth study of the cells found in nasopharyngeal swabs may allow to better characterize inflammation and innate immunity cells recruitment profiles according to viruses, codetections, and age of patients. The study of gene expression and inflammation markers, such as cytokines, would also provide insights on the differences between viruses, regarding pathways activated following infections.

We performed HRV and RSV VL quantitation. HRV VL was higher in the elderly than in the other age classes. A study published in 2015 (Lee et al., 2015) including adults hospitalized for RSV infection found a strong association between the level of RSV VL and the severity of ARI, but the median age of patients was 78 years old. This could suggest a decrease of innate immunity defenses in the elderly, which is described in the literature (Cunha, 2001). Concerning the RSV, we compared VL in hospitalized infants (Guérande) and infants in primary care (ECOVIR): RSV-A VL was higher in hospitalized infants, and RSV-B VL was not different in both cohorts. HRV VL was higher in infants seen in primary care. In our hospital cohort, HRV was co-detected in 93% of cases, mostly with RSV. We could explain this lower HRV VL in hospitalized infants by the putative negative interference between HRV and RSV. When co-detected with RSV, HRV VL is lower, suggesting that these viruses probably use similar mechanisms during infection. While HRV VL appear to be higher alone than in co-infection, this trend is based on 3 samples only and should not be considered as is. More samples are required to better characterize the effect of monoinfection VS co-infection regarding HRV VL. Moreover, the higher VL of RSV-A in the presence of HRV may indicate that RSV-A could benefit from HRV coinfection. Intriguingly, the opposite is observed (RSV-A VL decrease) when RSV-B is found in addition to RSV-A + HRV coinfection. This may suggest a trade-off between these viruses, according to which despite a global negative interference, RSV-A replication in airways may benefit from a coinfection with HRV, while not in the presence of RSV-B, but this last assumption requires to be confirmed by more observations of HRV + RSV-A + RSV-B coinfections or cellular model experiments. These observations unravel complex interactions between these viruses and would need further in-depth investigations to be fully understood.

Most studies did not show an association between HRV VL and ARI severity. A study published in 2017 compared viral loads in ambulatory patients versus intensive care patients: the HRV viral loads were not significantly different between these two patient populations (Granados et al., 2017). VL were similar across age groups (all age groups were represented). This is not what we found in our cohorts: HRV VL were higher for the three serotypes (HRV-A, HRV-B and HRV-C) in primary care compared to hospitalized infants. This is reminiscent of the possible negative interference in coinfections (HRV/RSV) affecting HRV VL. A Japanese study showed an association between viral load and ARI severity in children between 11 months and 3 years of age (Takeyama et al., 2012). This was no longer observed after the age of 4 years, nor before the age of 11 months. HRV VL seem to vary according to age and severity of the patients.

In an attempt to characterize the VL temporal dynamics, HRV and RSV were compared according to the delay between the first symptoms presented by the patients. We found no difference for both viruses. Personal differences in susceptibility and perception could explain these results. These results were not obtained from VL repeatedly obtained from a single patient during the course of infection but instead from a pool of patients sampled one time each and this represents a severe bias against accurate viral load dynamics estimation. A follow-up of single patients with repeated VL measurement during a viral ARI would be the best way to establish a “natural history” of the viral ARIs but represents a challenge in term of acceptation and ethics.

At last, respiratory microbiota is different in every patient, depending on age, personal history, and other factors. From early childhood, the respiratory microbiota determines respiratory health, and therefore repeated viral infections (de Steenhuijsen Piters et al., 2020). Studying the potential link between respiratory microbiota and viral coinfections could also be of interest to better understand the future respiratory health of our young patients.

## 5 Conclusion

By comparing the prevalence of viruses in different settings and quantifying respiratory viral loads of the most abundant viruses found in codetection in ARI (HRV and RSV), we know that health conditions, virus type, patient age and presence or absence of viral coinfections matter. This article highlights the complex relationship between HRV and RSV and suggests in many different cases that when in co-infection with RSV, HRV replicates at a lower level, confirming competition and a negative interference between these two viruses. Despite this negative interference for HRV, RSV-A benefits from this coinfection with HRV alone by an unknown mechanism. In addition to these virus-virus interactions, the interplay between host innate immunity and viruses probably plays a central role in these interactions.

## Data availability statement

The datasets presented in this study can be found in online repositories. The names of the repository/repositories and accession number(s) can be found below: https://datadryad.org/stash, DOI: 10.5061/dryad.bvq83bkgb.

## Ethics statement

The studies involving humans were approved by “Est II” protection committee (study reference 18/10/10/63004). The studies were conducted in accordance with the local legislation and institutional requirements. Written informed consent for participation in this study was provided by the participants’ legal guardians/next of kin.

## Author contributions

HP: Conceptualization, Data curation, Formal analysis, Funding acquisition, Investigation, Methodology, Project administration, Resources, Software, Supervision, Validation, Visualization, Writing – original draft. SC: Investigation, Methodology, Writing – review & editing. BL: Formal analysis, Visualization, Writing – review & editing. AV: Conceptualization, Funding acquisition, Supervision, Validation, Writing – review & editing. MA: Conceptualization, Formal analysis, Funding acquisition, Methodology, Supervision, Validation, Writing – review & editing.
